# Legionnaires’ disease in Europe, 2011 to 2015

**DOI:** 10.2807/1560-7917.ES.2017.22.27.30566

**Published:** 2017-07-06

**Authors:** Julien Beauté, on behalf of the European Legionnaires’ Disease Surveillance Network

**Affiliations:** 1 European Centre for Disease Prevention and Control (ECDC), Stockholm, Sweden; 2 Department of Medical Epidemiology and Biostatistics, Karolinska Institutet, Stockholm, Sweden; 3 The members of the network are listed at the end of the article

**Keywords:** Legionnaires' disease, surveillance, Epidemiology, Europe

## Abstract

Under the coordination of the European Centre for Disease Prevention and Control (ECDC), the European Legionnaires’ disease Surveillance Network (ELDSNet) conducts surveillance of Legionnaires’ disease (LD) in Europe. Between 2011 and 2015, 29 countries reported 30,532 LD cases to ECDC (28,188 (92.3%) confirmed and 2,344 (7.7%) probable). Four countries (France, Germany, Italy and Spain) accounted for 70.3% of all reported cases, although their combined populations represented only 49.9% of the study population. The age-standardised rate of all cases increased from 0.97 cases/100,000 population in 2011 to 1.30 cases/100,000 population in 2015, corresponding to an annual average increase of 0.09 cases/100,000 population (95%CI 0.02–0.14; p = 0.02). Demographics and infection setting remained unchanged with ca 70% of cases being community-acquired and 80% occurring in people aged 50 years and older. Clinical outcome was known for 23,164 cases, of whom 2,161 (9.3%) died. The overall case fatality ratio decreased steadily from 10.5% in 2011 to 8.1% in 2015, probably reflecting improved reporting completeness. Five countries (Austria, Czech Republic, Germany, Italy, and Norway) had increasing age-standardised LD notification rates over the 2011−15 period, but there was no increase in notification rates in countries where the 2011 rate was below 0.5/100,000 population.

## Background

Legionnaires’ disease (LD) is a severe pneumonia caused by Gram-negative bacteria, *Legionella* spp., which are found in freshwater environments worldwide and tend to contaminate man-made water systems [1]. People are infected by inhalation of contaminated aerosols and person-to-person transmission is exceptional [2]. LD is notifiable in all 30 European Union and European Economic Area (EU/EEA) countries, which reported 5,500 to 6,500 LD cases annually between 2005 and 2010 with annual age-standardised rates fluctuating around one LD case per 100,000 inhabitants [3]. This overall rate masked important differences across countries, with rates far below the European average reported by eastern and south-eastern European countries [4]. The notification rate was higher in males and increased with age. Approximately 70% of all reported cases were community-acquired, 20% travel-associated and 10% healthcare-related [2].

Several factors could possibly contribute to an increase of notified LD cases in Europe from 2011 to 2015. Previous studies have suggested that environmental conditions, especially rainfall and temperature, can affect the incidence of sporadic community-acquired LD cases [5,6] and it has been predicted that climate change will result in increases in temperature and changes in rainfall in Europe [7]. Additionally, the population structure of the EU/EEA is changing, with an increasing proportion of older persons who are more at risk of LD. People aged 65 years and over accounted for 16.4% of the total EU/EEA population in 2004 and for 18.5% in 2014 [8]. Finally, surveillance of LD in some European countries may be improving, as suggested by an evaluation carried out in France [9].

The objective of this study was to describe the epidemiology of LD in the EU/EEA from 2011 to 2015 and identify potential changes or trends in LD notification.

## Methods

Under the coordination of the European Centre for Disease Prevention and Control (ECDC), the European Legionnaires’ disease Surveillance Network (ELDSNet) conducts surveillance of LD in Europe. ELDSNet includes all 28 EU Member States, plus Iceland and Norway. Since 2010, nominated ELDSNet members in each of the participating countries have annually reported all LD cases that fulfil the EU case definition [10] to the European Surveillance System (TESSy) database hosted by ECDC. The EU case definition was amended in 2012 to the effect that probable cases should be reported with at least one positive laboratory test for a probable case. All cases reported during the years from 2011 to  2015 and meeting the 2012 EU/EEA case definition of confirmed and probable cases were included in the analysis. This study excluded Croatia from the analysis, because it only started reporting LD in 2013.

Information retrieved from TESSy included age, sex, date of disease onset, probable setting of infection, cluster status, laboratory method used for diagnosis, pathogen and clinical outcome. This study used population denominator data provided by the Statistical Office of the European Union (Eurostat) for calculating rates [11].

Continuous variables were compared across strata with the Mann–Whitney U test. Categorical variables were compared using chi-squared or Fisher exact tests. Age-standardised rates (ASR) were calculated using the direct method and the average age structure of the EU/EEA population for the period 2000 to 2010. Crude and age-adjusted male-to-female rate ratios were calculated. To test for trend, a linear regression was fitted to age-standardised notification rates over the 2011–15 period [12]. Goodness of fit of linear regressions was assessed using F statistics. Stata software release 14 (StataCorp. LP, US) was used for all data management and statistical analyses.

## Results

### Case classification and notification rate

**Figure 1:**
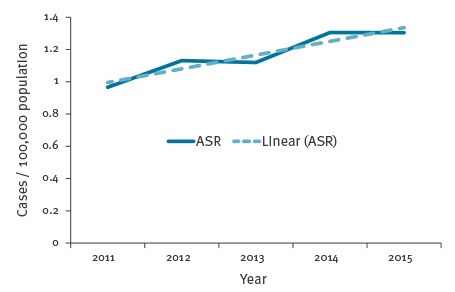
Age-standardised rate of Legionnaires’ disease per 100,000 population, European Union/European Economic Area, 2011–2015

Over the 2011–15 period, 29 countries reported 30,532 LD cases to ECDC, of which 28,188 (92.3%) were confirmed cases and 2,344 (7.7%) probable cases. The proportion of confirmed cases increased from 90.6% in 2011 to 93.3% in 2015. In 2011, fewer than 50% of cases reported by Latvia (38%), Poland (44%), and Romania (0%) were confirmed. These three countries had more than 70% of their cases confirmed in 2015. The annual number of reported cases ranged from 4,915 in 2011 to 6,986 in 2015. The ASR increased from 0.97 cases per 100,000 population in 2011 to 1.30 cases per 100,000 population in 2015, which corresponds to an annual average increase of 0.09 cases per 100,000 population (95% confidence interval (CI) 0.02–0.14; p = 0.02). (Figure 1).

ASR: age-standardised rate.

This study excluded Croatia from the analysis, because it only started reporting Legionnaires’ disease in 2013.

### Geographical distribution

Four countries (France, Germany, Italy and Spain) accounted for 70.3% of all reported cases, although their combined populations represented only 49.9% of the study population (Table 1)

**Table 1:** Number of reported cases of Legionnaires’ disease and age-standardised rates per 100,000 population, by reporting country and year, European Union/European Economic Area, 2011–2015

Country	2011		2012		2013		2014		2015	
	Number	ASR	Number	ASR	Number	ASR	Number	ASR	Number	ASR
Austria	96	1.12	104	1.22	100	1.13	133	1.48	160	1.79
Belgium	79	0.71	84	0.45	155	1.37	200	1.72	196	1.44
Bulgaria	0	0.00	0	0.00	1	0.01	1	0.01	1	0.01
Cyprus	1	0.13	7	1.01	6	0.76	6	0.80	2	0.25
Czech Republic	57	0.52	56	0.53	67	0.63	110	1.03	120	1.10
Denmark	123	2.20	127	2.29	113	2.02	158	2.76	185	3.24
Estonia	7	0.51	3	0.24	10	0.76	8	0.59	6	0.43
Finland	9	0.16	10	0.18	15	0.24	10	0.17	17	0.27
France	1,170	1.84	1,298	2.01	1,262	1.92	1,348	2.04	1,389	2.07
Germany	635	0.73	628	0.72	810	0.90	832	0.92	865	0.95
Greece	18	0.16	29	0.25	38	0.33	27	0.24	29	0.25
Hungary	37	0.36	33	0.32	29	0.29	32	0.32	58	0.56
Iceland	3	1.35	2	0.71	0	0.00	4	1.30	1	0.36
Ireland	6	0.18	15	0.44	14	0.39	8	0.20	11	0.30
Italy	1,021	1.56	1,346	2.04	1,363	2.04	1,510	2.21	1,556	2.23
Latvia	49	2.32	48	2.33	34	1.63	38	1.86	22	1.09
Lithuania	2	0.07	9	0.31	1	0.04	8	0.28	7	0.25
Luxembourg	6	1.20	5	1.04	7	1.27	5	0.91	5	0.91
Malta	9	2.07	4	1.04	2	0.35	9	1.79	6	1.38
The Netherlands	311	1.88	304	1.83	308	1.83	348	2.04	419	2.39
Norway	33	0.72	25	0.52	40	0.83	51	1.06	60	1.22
Poland	18	0.05	8	0.02	11	0.03	12	0.03	23	0.06
Portugal	89	0.82	140	1.28	94	0.85	588	5.33	145	1.30
Romania	1	0.00	3	0.02	1	0.00	1	0.00	5	0.03
Slovakia	7	0.13	4	0.08	6	0.12	14	0.26	14	0.27
Slovenia	44	2.14	81	3.84	77	3.62	59	2.76	106	4.98
Spain	706	1.52	972	2.07	815	1.72	925	1.78	1,024	2.12
Sweden	127	1.32	102	1.04	122	1.25	136	1.38	142	1.42
United Kingdom	251	0.41	401	0.66	331	0.54	370	0.59	412	0.65
EU/EEA	4,915	0.97	5,848	1.13	5,832	1.12	6,951	1.31	6,986	1.30

ASR: age-standardised rate; EEA: European Economic Area; EU: European Union.

This study excluded Croatia from the analysis, because it only started reporting Legionnaires’ disease in 2013.

**Figure 2:**
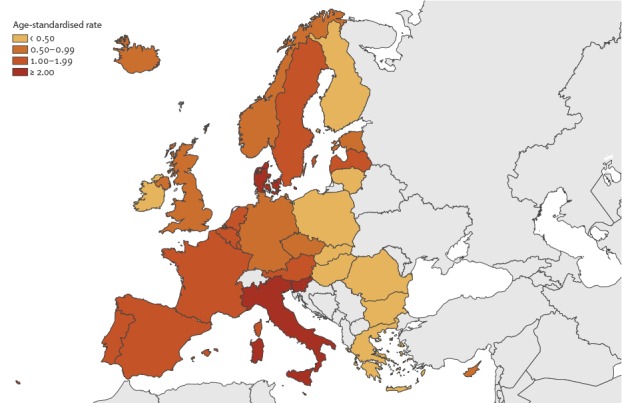
Age-standardised rate of Legionnaires’ disease per 100,000 population by country, European Union/European Economic Area, 2011–2015

Conversely, the 20 lowest reporting countries reported only 10.2% of all cases, although their combined populations represented 28.8% of the study population. Country-specific average ASR ranged from 0.01 cases per 100,000 population in Bulgaria and Romania to 3.46 cases per 100,000 population in Slovenia (Figure 2).

This study excluded Croatia from the analysis, because it only started reporting Legionnaires’ disease in 2013.

Denmark, Italy, and Slovenia had ASR above 2.00 cases per 100,000 population. Over the 2011–15 period, five countries had significant trends: ASR increased in Austria (0.16 additional cases per 100,000 population each year, 95% CI: 0.00 to 0.32), Czech Republic (0.16, 95% CI: 0.05 to 0.28), Germany (0.06, 95% CI: 0.01 to 0.12), Italy (0.15, 95% CI: 0.00 to 0.30), and Norway (0.15, 95%CI: 0.01 to 0.30). In Latvia, the ASR decreased by 0.29 cases per 100.000 each year (95%CI -0.57 to -0.02). Portugal and Slovenia observed the highest yearly ASR with 5.33 cases per 100,000 population in 2014 and 4.98 cases per 100,000 population in 2015, respectively (Table 1).

### Age and sex

Information on age was available for 30,462 (99.8%) cases, of which 24,353 (79.8%) were 50 years old or older (Table 2).

**Table 2:** Main characteristics of reported Legionnaires’ disease cases, European Union/European Economic Area, 2011−2015

Characteristics	Cases		Notification rate/100,000 population
	Number	Percentage (%)	
All cases	30,532	100	1.21
Age group (years)			
< 20	159	0.5	0.03
20–29	473	1.6	0.15
30–39	1,440	4.7	0.41
40–49	4,037	13.3	1.08
50–59	6,917	22.7	2.00
60–69	7,120	23.4	2.52
70–79	5,882	19.3	2.88
≥ 80	4,434	14.6	3.47
Unknown	70	NA	NA
Sex			
Male	21,618	71.1	1.75
Female	8,789	28.9	0.68
Unknown	125	NA	NA
Probable setting of infection			
Community	19,019	70.7	0.75
Travel abroad	3,098	11.5	NA
Domestic travel	2,259	8.4	NA
Nosocomial	1,322	4.9	NA
Other healthcare	651	2.4	NA
Other	551	2.0	NA
Unknown	3,632	NA	NA
Cluster status			
Sporadic cases	19,559	90.1	NA
Clustered cases	2,158	9.9	NA
Unknown	8,815	NA	NA
Outcome			
Alive	21,003	90.7	NA
Dead	2,161	9.3	0.09
Unknown	7,368	NA	NA

NA: Not applicable.

This study excluded Croatia from the analysis, because it only started reporting Legionnaires’ disease in 2013.

**Figure 3:**
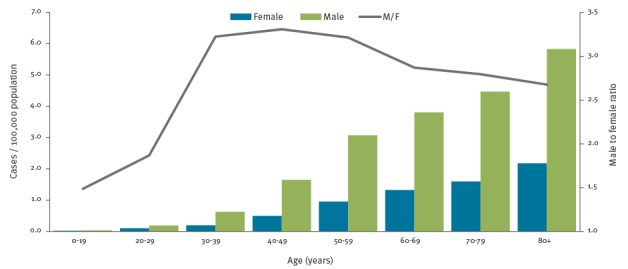
Notification rates of Legionnaires’ disease per 100,000 population by sex and age group and male-to-female rate ratio by age group, European Union/European Economic Area, 2011–2015

Notification rates increased with age in both sexes, peaking at 5.8 cases per 100,000 population in males aged 80 years or older (Figure 3).

This study excluded Croatia from the analysis, because it only started reporting Legionnaires’ disease in 2013.

LD was more common in males with a crude male-to-female rate ratio of 2.6:1 (age-adjusted 2.9:1). This ratio, which was stable over the 2011–15 period, steeply increased with age from 1.5:1 below 20 years to 3.3 in those aged 40–49 years and then slowly decreased in older age groups (Figure 3). Age-adjusted male-to-female rate ratio ranged from 1.1:1 in Slovakia to 5.0:1 in Cyprus. At date of onset, females (median 66 years, interquartile ratio (IQR): 54–77) were older than males (median 61 years, IQR: 51–72) (p < 0.01).

### Probable setting of infection

The probable setting of infection acquisition was known for 26,900 cases reported from 2011 to 2015. Of these, 19,019 (70.7%) were reported as community-acquired, 5,357 (19.9%) as travel-associated, 1,973 (7.3%) as healthcare-related and 551 (2.0%) as associated with other settings (Table 2). The distribution of cases by setting of acquisition of infection remained stable over the study period. Of 16 countries that reported at least 80% of their cases with known probable setting of infection, the proportion of community-acquired cases ranged from 38.8% in Norway to 96.1% in Slovenia. Norway had the highest proportion of cases associated with a stay abroad (61.2%) while the same proportion was below 2% in Bulgaria, Italy, Latvia, and Spain.

### Outcome

Clinical outcome was known for 23,164 cases, of which 2,161 (9.3%) died (Table 2). The case fatality ratio did not differ significantly by sex (9.1% in males vs 9.9% in females, p = 0.06). Over the 2011−15 period, the overall case fatality ratio decreased continuously, from 10.5% in 2011 to 8.1% in 2015. Over the same time, the proportion of cases reported with unknown outcome has continuously decreased from ca 30% in 2011 to 20% in 2015, and the crude mortality rate fluctuated between 0.07 and 0.09 deaths per 100,000 population.

### Laboratory tests and pathogens

Over the 2011−15 period, 33,809 laboratory tests were recorded for the 30,532 reported LD cases, of which 78.2% were urinary antigen tests (UAT), 10.8% cultures, 6.8% PCR and the remaining 1.1% other tests. Eleven of the 29 reporting countries reported more than one laboratory test for some of their cases. The proportion of cases diagnosed by UAT was 78.9% in 2011 and fluctuated between 87.3% and 88.9% from 2012 to 2015. The proportion of culture-confirmed cases was stable over the period at ca 12%. The proportion of cases reported to have been diagnosed by PCR increased from 4.3% in 2011 to 10.5% in 2015. Conversely, whereas 5.5% of cases were ascertained on the basis of a single high titre of a specific serum antibody in 2011, this proportion decreased to less than 2% in 2015. Similarly, the proportion of cases reported on the basis of a fourfold rise in titre decreased steadily from 1.6% in 2011 to 0.5% in 2015. Only 17 cases (< 0.1%) were reported as diagnosed by direct immunofluorescence during the 2011−15 period. Of the 3,645 culture-confirmed cases reported, 3,511 (96.3%) were due to *Legionella pneumophila*, including 3,020 (82.9% of culture-confirmed cases) due to *L. pneumophila* serogroup 1 (Table 3). This proportion was stable over the study period.

**Table 3:** Reported culture-confirmed cases of Legionnaires’ disease and *Legionella pneumophila* isolates by species and serogroup, European Union/European Economic Area, 2011–2015 (n=3,645)

Species	Serogroups	Culture-confirmed cases	
		No.	%
*Legionella pneumophila*	serogroup 1	3,020	82.9
	serogroup 2	19	0.5
	serogroup 3	101	2.8
	serogroup 4	13	0.4
	serogroup 5	19	0.5
	serogroup 6	42	1.2
	serogroup 7	9	0.2
	serogroup 8	8	0.2
	serogroup 9	5	0.1
	serogroup 10	19	0.5
	serogroup 11	3	0.1
	serogroup 12	1	< 0.1
	serogroup 13	2	0.1
	serogroup 14	7	0.2
	serogroup mixed	4	0.1
	non-serogroup 1	7	0.2
	serogroup unknown	232	6.4
*L. anisa*	NA	2	0.1
*L. bozemanii*	NA	15	0.4
*L. cincinnatiensis*	NA	1	< 0.1
*L. dumoffii*	NA	2	0.1
*L. longbeachae*	NA	35	1.0
*L. macaechernii*	NA	1	< 0.1
*L. micdadei*	NA	12	0.3
*L. sainthelensi*	NA	1	0.0
*L.* other species	NA	27	0.7
*L.* species unknown	NA	38	1.0
Total	NA	3,645	100

This study excluded Croatia from the analysis, because it only started reporting on Legionnaires’ disease in 2013.

### Clusters

Of the 21,717 cases reported with known cluster status, 19,559 (90.1%) were reported as sporadic cases (Table 2). Over the study period, 19 countries reported 2,158 cases as part of a cluster. Of these 2,158 cases, 1,923 (89.1%) were reported by six countries (Germany, Italy, the Netherlands, Portugal, Spain, and the United Kingdom (UK)). Of the 2,090 clustered cases with known probable setting of infection, 1,050 (50.2%) were reported as community-acquired, 841 (40.2%) as travel-associated, 150 (7.2%) as healthcare-related and 49 (2.3%) as associated with other settings. Of the 2,158 clustered cases, 1,348 (62.5%) were reported with a cluster identifier. The five largest reported clusters were in Portugal (403 cases in 2014 and 30 cases from 2011 to 2013), Spain (39 and 18 cases in 2012), and the UK (23 cases in 2012).

## Discussion

After the peak observed in 2010, the LD notification rate in the EU/EEA in 2011 returned to the levels observed from 2005 to 2009, when one case per 100,000 population was reported each year [3]. The reasons behind the unexpected increase observed in 2010 were not investigated in all countries, but a study in the Netherlands strongly suggested an association with warm and wet weather during the summer [13]. Over the 2011−15 period, the EU/EEA age-adjusted notification rate steadily increased to reach 1.30 case per 100,000 population in 2014−15, the highest rate ever observed. Active surveillance of legionellosis in the United States found a comparable incidence of 1.30 per 100,000 population from 2011 to 2013, but included notifications of both LD and Pontiac fever [14]. The overall ASR increase in the EU/EEA over the 2009−15 period would suggest an effect beyond demographic change. It is also possible that the study period was too short to detect the effect of an ageing population in Europe. This overall increase was probably mostly driven by increases observed in a few populous countries, such as Germany and Italy.

In the five countries with ASR increasing trend over the 2011−15 period, the increase was progressive which would be consistent with an improvement in surveillance, although our data cannot exclude an effect of weather conditions more favourable to the growth of *Legionella* in some regions during the study period. Such improvement has already been reported in Italy over the 2000−11 period [15]. In other countries with stable ASR, the increasing number of LD cases would be compatible with an effect of demographic change with increasing numbers of at-risk older people. In Latvia, the decreasing ASR may reflect an improvement in laboratory ascertainment with less reliance on serological testing [16] and an increasing use of UAT, which is more specific.

None of the countries with notification rates below 0.5 cases per 100,000 population had substantially increasing ASR over the period studied. Reasons behind this possible under-ascertainment are probably multiple and country-specific. A study using travel-associated Legionnaires’ disease case notification and tourism denominator data strongly suggested substantial under-ascertainment in Greece [17]. The report of the first cluster of travel-associated Legionnaires’ disease detected in Bulgarian residents pointed out the lack of diagnostic tests on site and the lack of requests for legionella microbiology from physicians [18]. Similarly, lack of awareness and underdiagnoses were reported in Poland [19]

From 2011 to 2015, large outbreaks of LD were documented in Germany, Portugal, Spain, and the UK [20-24]. Our data captured some of these, such as the community outbreak that occurred in Vila Franca de Xira, Portugal in 2014 [23], the community outbreak in Edinburgh, UK in 2012 [21] or the travel-associated outbreak in Calp, Spain in 2012 [20]. With the notable exception of the Vila Franca de Xira outbreak, the number of cases reported associated with these clusters only accounted for a small proportion of the cases reported at the country level. Several small clusters (2–3 cases) of travel-associated cases were reported, which might reflect both a higher probably of clustering in a travel setting and a result of the near-real-time surveillance of travel-associated cases within ELDSNet [25].

The shape of the male-to-female rate ratio by age group is suggestive of a higher male bias among adults aged 30–59 years. Given the severity of LD, differences in health-seeking behaviour between sexes are unlikely. This could be explained by behavioural differences between sexes across age groups, such as smoking habits. Smoking prevalence is decreasing in most EU/EA countries among both sexes, with a steeper decrease in males [26]. Sex smoking differences are narrowing but consequences may only be perceived in future cohorts of LD cases. Exposure to other risk factors such as home plumbing work could be associated with sex and age [27]. Unfortunately, known risk factors for LD such as smoking or comorbidities are not collected as part of routine surveillance at EU/EEA level. Such factors might partly explain sex, age or country differences with heterogeneous burden of chronic pulmonary disease across Europe [28]. In addition, a recent study carried out in New York suggested that occupation and other social determinants may also represent risks factors for LD [29]. Although only a few LD cases were under 20 years of age, LD should not be overlooked in children, especially those with underlying conditions such as cancer [30].

The decreasing case fatality ratio may suggest a reporting bias towards fatal outcomes in the past which is gradually being corrected by improved reporting completeness. The main demographic characteristics and probable settings of infection of LD cases remained unchanged during the study period and comparable to previous reports [3].

The landscape of laboratory tests used to diagnose LD has changed over the past years. If both UAT and culture have remained the most frequently used tests, the late 2000s have confirmed the rise of PCR and the inexorable decline of diagnosis by fourfold titre rise and single high titre in specific serum antibody [25]. The main limitation of UAT is its poor sensitivity to non-*L. pneumophila* serogroup 1 strains [31]. The number of culture-confirmed cases reported with an infection caused by other species should remind us the risk of an exclusive reliance on UAT.

Trends observed at national level may mask sub-national disparities, especially in large countries. This has been documented in Italy where a study has suggested higher under-reporting in central and southern regions compared with northern regions [15]. This analysis could not capture such heterogeneity, but these findings could be highly valuable for informing national policies.

These findings illustrate the added-value of the network whose activities help ensure the collection of high-quality data. All reporting countries use the same reporting protocol and case definition. In addition, ELDSNet offers training and external quality assessment (EQA) to improve laboratory capabilities.

## Conclusion

The burden of LD appears to be growing in Europe and at least 450 people still die of LD each year in the EU/EEA. The epidemiology is very similar to that observed in the United States, with a comparable notification rate and similar settings of infection. In countries with persistently low notification rates, ad hoc studies should identify reasons for under-ascertainment. All countries should endeavour to develop and maintain appropriate control measures in man-made water systems to prevent LD cases.

## Notes

**The European Legionnaires’ disease Surveillance Network:** Daniela Schmid, Günther Wewalka (Austria); Olivier Denis, Stéphanie Jacquinet, Denis Piérard, Sophie Quoilin (Belgium); Iskra Tomova (Bulgaria); Despo Pieridou, Ioanna Gregoriou, Maria Koliou (Cyprus); Vladimir Drašar, Irena Martinkova (Czech Republic); Charlotte Kjelsø, Søren Anker Uldum (Denmark); Irina Dontsenko, Rita Peetso (Estonia); Sari Jaakola; Jaana Kusnetsov; Outi Lyytikäinen; Silja Mentula (Finland); Christine Campese, Sophie Jarraud, Agnes Lepoutre (France); Bonita Brodhun, Christian Lück (Germany); Georgia Spala (Greece); Ágnes Fehér, Ildikó Ferenczné Paluska (Hungary); Thorolfur Gudnason, Guðrún Sigmundsdóttir (Iceland); Mary Hickey, Tara Mitchell, Joan O’Donnell, Darina O’Flanagan (Ireland); Maria Grazia Caporali, Maria Luisa Ricci, Maria Cristina Rota (Italy); Antra Bormane, Jelena Galajeva, Oksana Savicka (Latvia); Migle Janulaitiene, Simona Zukauskaite-Sarapajeviene (Lithuania); Paul Reichert (Luxembourg); Jackie Maistre Melillo, Tanya Melillo Fenech, Graziella Zahra (Malta); Petra Brandsema, Ed IJzerman, Leslie Isken, Daan Notermans, (Netherlands); Dominique Caugant, Heidi Lange (Norway); Michal Czerwinski, Katarzyna Piekarska (Poland); Teresa Fernandes, Maria Teresa Marques (Portugal); Daniela Badescu, Gratiana Chicin (Romania); Danka Šimonyiová, Margita Špaleková (Slovak Republic); Maja Sočan, Darja Kese (Slovenia); Rosa Cano-Portero, Carmen Pelaz Antolin (Spain); Margareta Löfdahl (Sweden); Eleanor Anderson; Tim Harrison; Falguni Naik; Nick Phin; Kevin Pollock; Alison Potts; Elaine Stanford (United Kingdom)
