# Authors’ correction for Euro Surveill. 2017;22(27)

**DOI:** 10.2807/1560-7917.ES.2017.22.46.171116-1

**Published:** 2017-11-16

**Authors:** Eurosurveillance editorial team

**Affiliations:** 1 European Centre for Disease Prevention and Control (ECDC), Stockholm, Sweden

In the article ‘Legionnaires’ disease in Europe, 2011 to 2015’, published on 6 July 2017, the percentage of ‘other tests’ in the first sentence in the results section ‘Laboratory tests and pathogens’, was erroneously 1.1%. The correct number is 4.2%. On request of the authors, the manuscript was updated accordingly on 14 November 2017.

